# “I have got something positive out of this situation”: psychological benefits of caregiving in relatives of young people with muscular dystrophy

**DOI:** 10.1007/s00415-013-7176-8

**Published:** 2013-11-08

**Authors:** Lorenza Magliano, Melania Patalano, Alessandra Sagliocchi, Marianna Scutifero, Antonella Zaccaro, Maria Grazia D’Angelo, Federica Civati, Erika Brighina, Giuseppe Vita, Gian Luca Vita, Sonia Messina, Maria Sframeli, Marika Pane, Maria Elena Lombardo, Roberta Scalise, Adele D’Amico, Giulia Colia, Michela Catteruccia, Umberto Balottin, Angela Berardinelli, Maria Chiara Motta, Corrado Angelini, Alessandra Gaiani, Claudio Semplicini, Luca Bello, Roberta Battini, Guja Astrea, Giulia Ricci, Luisa Politano

**Affiliations:** 1 Department of Psychology, Second University of Naples (SUN), Viale Ellittico, 31, 81100 Naples, Caserta Italy; 2 Cardiomyology and Medical Genetics, Department of Experimental Medicine, Second University of Naples (SUN), Naples, Italy; 3 NeuroMuscular Unit, Department of NeuroRehabilitation, IRCCS “E. Medea”, Bosisio Parini (LC), Italy; 4 Department of Neurosciences, University of Messina, Messina, Italy; 5 “Nemo Sud” Clinical Centre, University Hospital of Messina, Messina, Italy; 6 Department of Paediatric Neurology, Catholic University, Rome, Italy; 7 Unit of Neuromuscular and Neurodegenerative Diseases, Bambin Gesù Children’s Hospital, Rome, Italy; 8 Child Neuropsychiatry Unit, Department of Brain and Behavioral Sciences, University of Pavia, Pavia, Italy; 9 Child Neuropsychiatry Unit, “C. Mondino” National Neurological Institute, Pavia, Italy; 10 Department of Neurosciences, University of Padova, Padua, Italy; 11 IRCSS San Camillo, Lido, Venice, Italy; 12 Developmental Neuroscience, IRCCS Stella Maris, University of Pisa, Pisa, Italy

**Keywords:** Muscular dystrophy, Psychological benefits, Caregiving, Social network, Professional support

## Abstract

This paper focuses on the psychological benefits of caregiving in key relatives of patients with muscular dystrophies (MD), a group of rare diseases characterized by progressive weakness and restriction of the patient’s functional abilities. We describe whether relatives perceived caregiving to be a positive experience and test whether relatives’ perceptions vary in relation to their view of the patient as a valued person, the degree of involvement in care, and the level of support provided by social network and professionals. The study sample included 502 key relatives of patients aged 4–25 years, suffering from Duchenne, Becker, or limb-girdle MD, in treatment for at least 6 months to one of the eight participating centers, living with at least one relative aged 18–80 years. Of key relatives, 88 % stated that they had gotten something positive out of the situation, 96 % considered their patients to be sensitive, and 94 % viewed their patients as talented. Positive aspects of caregiving were more recognized by key relatives who were more convinced that the patient was sensitive and who perceived that they received higher level of professional help and psychological social support. These results suggest that most key relatives consider that their caregiving experience has had a positive impact on their lives, despite the practical difficulties of caring for patients with MD. Professionals should help relatives to identify the benefits of caregiving without denying its difficulties. Clinicians themselves should develop positive attitudes towards family involvement in the care of patients with long-term diseases.

## Introduction

Research and clinical practice highlight that family involvement during the care of patients with long-term illnesses facilitates the patient’s acceptance of the disease [1, 2], improves compliance to the therapeutic program [3, 4], and beneficially influences the patient’s clinical response to treatment [5]. For the relatives, long-term assistance of the patient may be a both demanding and rewarding experience [6–10].

Though the difficulties experienced by caregiving relatives have been extensively investigated [11–16], the positive aspects of family care have scarcely been explored. The limited data regarding the benefits of caregiving are mainly derived from studies of relatives of patients with dementia [17, 18] and cancer [19–22]. These data reveal that a sense of personal growth [9, 19, 23], appreciation from the care receiver [24], family closeness, the strength to face new challenges, a day-to-day approach to life [19, 21], spirituality [19–21, 25, 26], and reprioritization of values [20, 21] are psychological consequences frequently experienced by caregiving relatives [18, 20, 21]. Studies on this topic have also shown that relatives of patients with a higher level of family dependence more frequently acknowledge the positive aspects of caregiving [8]. The same has also been shown in relatives who felt adequately supported by both their social network and professionals [21, 26].

Although there are common elements across the spectrum of long-term illnesses that may contribute to relatives perceiving caregiving to be a rewarding experience, differences in perception exist between diseases. These differences mainly derive from the clinical characteristics and degree of social acceptance of each disease [11, 27]. Therefore, neither can data on caregiving by relatives be generalized, nor can one instance of caregiving by relatives speak for caregiving in general without first acknowledging the clinical characteristics or social acceptance of the disease in question.

In the case of muscular dystrophies (MD)—a group of rare diseases characterized by progressive weakness [28]—despite high levels of physical dependency, many patients live at home, where for several years they receive daily assistance from relatives [29, 30]. Data from a study by Pangalila et al. [6] of 80 parents of 57 adult patients with Duchenne MD (DMD) showed that 97 % of relatives felt that caring for their family member was important to them, while 90 % stated that caregiving was appreciated by the recipient. A study by Kenneson and Bobo [7] of 1,238 women who were caring for one or more relatives with DMD or Becker MD (BMD) found that 68 % of respondents felt mostly or totally satisfied with life. This study further revealed that respondents strongly associated social support with their relatives’ satisfaction with life. Even more interestingly, it found that caregiving women were more likely to have a spouse or cohabitant than women in the general population, which suggests that raising a child with a disability strengthened the social bond between partners. Findings from a qualitative study [31] on 12 parents of children with DMD found that relatives perceived the illness in different ways—as a severe loss, for example, or as a call to adapt or as a way to rediscover the child—and that their appraisals led to different strategies for coping with the illness.

In Italy in 2012, we performed a national survey regarding the condition of 502 families of patients aged 4–25 years who had a severe form of MD, either DMD, BMD, or limb-girdle MD (LGMD). The study aimed to describe the rewards and difficulties of the caregiving experience of key relatives, as well as the professional and social support on which they may rely. Caregiving was found to be a significantly greater psychological burden to families—considered as an indirect measure of stress—among relatives whose patients had a higher degree of disability, and among relatives who spent more hours caregiving per day, and who had poorer social support [32].

This paper focuses on the positive aspects of caregiving reported by the above-mentioned 502 key relatives of patients with MD. We describe whether relatives perceived caregiving to be a positive experience and test whether relatives’ perceptions vary in relation to their view of the patient as a valued person, the degree of involvement in care, and the level of support provided by the social network and professionals.

## Methods

### Study design

The study was carried out in Italy in eight treatment centers for MD from January to December of 2012. In each center, the key relatives (i.e., the relative spending more daily time in contact with the patient and being more involved in his or her care) of patients aged 4–25 years who had a diagnosis of DMD, BMD, or LGMD, who were in treatment for at least 6 months, and lived with at least one relative aged 18–80 years, were consecutively contacted and asked for their informed consent to participate in the study.

The protocol of the study was approved by the ethic committee of the Second University of Naples (coordinating center) and by the local ethical committee of each participating treatment center. The study has been performed in accordance with the ethical standards laid down in the 1964 Declaration of Helsinki and its later amendments.

On occasion of the patient’s scheduled clinical control, key relatives who have given their informed consent to participate in the study were interviewed regarding the patient’s level of functioning according to the Barthel index (BI), and were asked to fill in the Family Problems Questionnaire (FPQ) and the Social Network Questionnaire (SNQ) [33, 34].

### Assessment instruments

The FPQ explores the respondent’s burden, professional support, and social network support in emergencies concerning the patient. It also contains four items rated on a four-level scale (from “very much” to “not at all”) that focus on the key relatives’ perceptions of caregiving as a positive experience and of the patient as a sensitive and/or talented person. Two open-ended questions further asked key relatives to characterize the type of a patient’s talents or skills and, if the relative had reported positive aspects of caregiving, to elaborate upon those positive aspects. Based upon their content, the answers to the open-ended questions were grouped by two investigators (Melania Patalano and A.S.) into discrete categories. In particular, answers to positive aspects of caregiving were grouped into five categories: personal growth, altruism, resilience, sharing of experience, and other. Each patient’s talents or abilities were grouped into eight categories: technological, artistic, sporting, cognitive, manual, psychological, cultural, and other. Interrater reliability regarding use of these categories was measured using 50 randomly selected cases (Cohen’s *κ* value 0.90 and 0.98).

The SNQ explores the respondent’s social contact, level of practical and psychological social support, and quality of an intimate relationship with a partner. It also includes a question exploring whether the respondent’s social relationships were improved during the previous year. Further details on the design of the study, assessment procedures, and descriptions of the instruments are reported in a previous paper [32].

### Statistical analysis

The relationship of positive aspects of caregiving to the key relative’s sociodemographic characteristics (sex, age, marital status, occupation, level of education, and relationship with the patient), as well as the patient’s sociodemographic characteristics (sex, age, and level of education) and clinical variables (type of MD, duration of illness, and BI global score) were explored by analysis of variance (ANOVA) or Spearman’s *r* coefficient, as appropriate.

Correlations among positive aspects of caregiving and answers regarding relatives’ perception of the patient as a talented and sensitive person, the family’s burden, and its level of professional and social support were explored by Spearman’s *r* coefficient.

Hierarchical multiple regression was performed to study the simultaneous effects of the above-mentioned items on the relative’s recognition of positive aspects of caregiving. Only variables found to be statistically significantly related to positive aspects of caregiving on univariate analysis were included in the regression. Statistical significance was set at *p* < 0.05. Analyses were performed by using SPSS 19.0.

## Results

### Descriptive results

As shown in Table 1, the majority of the 502 patients were male, young, and in school. Sixty-six percent (333) of patients suffered from DMD, 26 % (129) from BMD, and 8 % (40) from LGMD. The mean level of independence in daily activities for patients, measured by the BI, was 68.3 (31.3 Standard Deviation, SD). Thirty-nine percent (194) of patients were in wheelchairs. Most patients were in drug treatments (73 %) and attended rehabilitation programs (67 %).

**Table 1:** Characteristics of the 502 patients and their key relatives

	Patients (*N* = 502)	Key relatives (*N* = 502)
Sex, *N* (%)		
Male	484 (96)	74 (15)
Female	18 (4)	428 (85)
Age, mean (SD) years	12.8 (5.6)	43.4 (7.4)
Marital status, *N* (%)		
Single	502 (100)	61 (12)
Cohabitant/spouse	0	441 (88)

	Attendance	Degree
Education, *N* (%) yes	430 (86)	502 (100)
Pre-school	50 (12)	–
Primary school	148 (34)	35 (7)
Secondary school	90 (21)	184 (37)
High school	127 (29)	219 (44)
University	17 (4)	64 (12)
Currently employed, *N* (%) yes	7 (1)	264 (53)
Relationship with the patient, %		
Mother	–	424 (84)
Father	–	70 (14)
Other	–	8 (2)
Duration of symptoms, mean (SD) years	8.9 (5.5)	–

Most of the 502 key relatives were mothers and lived with a partner or spouse. Almost half of key relatives had received higher education and were employed (Table 1). Twenty-two percent of key relatives received professional support, while 10 % were in contact with family and patient organizations.

Eighty-eight percent (434) of key relatives stated that they had gotten something positive out of their caregiving situation (Table 2), while 5 % (26) stated that they had considered parting from the patient in the past 2 months.

**Table 2:** Relatives’ acknowledgment of positive aspects of caregiving and of patient’s abilities

	Very much/a lot, *N* (%)	A little, *N* (%)	Not at all, *N* (%)
All things considered, I have got something positive out of this situation	326 (66)	108 (22)	58 (12)
P is sensitive and concerned about other people’s problems	275 (63)	142 (32)	22 (5)
I think that P has some special abilities or talents	339 (68)	141 (28)	20 (4)
In the past 2 months, the thought of parting from S crossed my mind	3 (1)	23 (4)	476 (95)

Among the 434 key relatives who reported that they had gotten something positive out of the situation, 374 (86 %) indicated at least one specific positive aspect of caregiving, which were grouped as follows:

- Personal growth (72 %) (e.g., “I learned to enjoy the little things” and “I learned that difficulties of life help you to grow”)
- Resilience (18 %) (e.g., “I learned to rely on myself,” “I learned to have more strength to fight for the people I love,” and “I learned to not lose heart”)
- Altruism (15 %) (e.g., “I get involved in helping people in a condition similar to mine” and “I became more sensitive to other people”)
- Sharing of the experience (3 %) (e.g., “I realized that I was not the only one to experience certain situations” and “I discovered that there are many people who are close to us”)
- Other (3 %) (e.g., “I discovered the importance of availability of physicians and of clinical follow-ups”)

Ninety-four percent (417) of key relatives considered their patients to be sensitive, while 96 % (480) considered their patients to be talented.

Among the 480 key relatives who mentioned their patient’s talents or abilities, 430 (90 %) specified the talents or abilities, which were grouped as follows:

- Psychological abilities (58 %) (e.g., “He is a playful boy. When we are down, he is able to keep our spirits up” and “He is a likeable child and he is loved by all”)
- Cognitive abilities (48 %) (e.g., “She is very smart, studious, and curious,” “He is a child with a lot of imagination and with excellent communication skills for science,” “He started to read during pre-school,” and “She is very intelligent and eager to learn”)
- Artistic abilities (23 %) (e.g., “He can sing and has a beautiful voice,” “She plays an instrument very well,” and “He draws and has won some prizes for his art”)
- Technological abilities (11.2 %) (e.g., “She is proficient with computers”)
- Cultural abilities (7 %) (e.g., “He loves to read,” “She likes history and to visit museums,” and “He likes music”)
- Sporting abilities (4 %) (e.g., “He loves to play soccer,” “She loves to swim,” and “He loves to go fishing”)
- Manual abilities (3 %) (e.g., “She excels in handicrafts, ceramics, and painting,” “He excels at cooking,” and “He enjoys carpentry”)
- Other (5 %) (e.g., “She has a passion for gardening,” “He loves trucks,” and “She’s an expert on sports”)

### Univariate and multivariate analyses

Positive aspects of caregiving were recognized with more conviction by key relatives who reported more practical difficulties (*r* = 0.14, *p* < 0.001) and by key relatives whose patients had a higher level of dependency (*r* = −0.16, *p* < 0.001) and a longer length of illness (*r* = 0.14, *p* < 0.003).

Perceiving the situation’s positive aspects was higher among relatives who were more convinced that their patient was a sensitive person (*r* = 0.20, *p* < 0.0001) and had talents and abilities (*r* = 0.20, *p* < 0.01). Furthermore, positive aspects were more often reported by key relatives who had more social contacts (*r* = 0.11, *p* < 0.01) and received support from their friends (*r* = 0.22, *p* < 0.0001) and/or partners (*r* = 0.14, *p* < 0.003), as well as by key relatives who reported having improved their social contacts during the previous year (*r* = 0.17, *p* < 0.0001). Finally, key relatives’ acknowledgment of positive aspects correlated with levels of professional support (*r* = 0.24, *p* < 0.0001).

Multivariate regression analysis accounted for 18 % of the variance in key relatives’ acknowledgment of positive aspects of caregiving (Table 3), confirming that these aspects were more recognized by key relatives who were more convinced that the patient was sensitive and who perceived that there was a higher level of professional help and psychological social support.

**Table 3:** Regression analysis: effects of patients’ clinical variables and relatives’ attitudes towards the patients, and social and professional support on acknowledgment of positive aspects of caregiving

	Block 1	Block 2	Block 3	Block 4
Independent variables	Standardized beta value			
BI global score	−0.10	−0.10	−0.10	−0.08
Objective burden	0.03	0.01	0.05	0.10
Duration of illness	0.07	0.04	0.04	0.06
Patient as sensitive person		0.17^c^	0.17^c^	0.16^c^
Patient abilities and talents		0.15^a^	0.13^a^	0.08
Professional help			0.21^d^	0.18^d^
Social contacts				−0.04
Support by the partner				0.02
Psychological support by the social network				0.20^c^
Improvement of social contacts in the last year				0.09
Model’s *F*, df, *p*<	8.5, 10, 394, 0.0001			
*R* ^2^	0.03	0.09	0.13	0.18

^a^ *p* < 0.01

^b^ *p* < 0.005

^c^ *p* < 0.001

^d^ *p* < 0.0001

## Discussion

### Positive aspects of caregiving and influential variables

This study shows that 84 % of the caregiving sample was represented by mothers and that few mothers admitted negative feelings in assisting their child, as expected taking into account Italian social customs.

The results of the study suggest that most key relatives consider that their caregiving experience has had a positive impact on their lives, despite the practical difficulties of caring for patients with MD.

In line with findings from other studies [14, 16, 20, 23], 88 % of key relatives reported that they had gotten something positive out of the situation. In particular, 72 % of key relatives mentioned having changed their perception of the meaning of life’s values, while 18 % mentioned an increased sense of strength and courage against adversities.

These findings can be interpreted within the framework of Lazarus and Folkman’s [35] transactional model, which postulates that an individual’s adaptation to an event is a process based on primary and secondary cognitive appraisal. In regards to MD, primary appraisal refers to the realization of what the illness is, while secondary appraisal implies the development of emotional and problem-oriented strategies to cope with the difficulties of caring for patients with MD. In this model, adaptation is significantly influenced by internal factors, such as the key relative’s attitude toward the patient, and external resources, such as the availability of social and professional support [36–38].

Key relatives’ intentions to be engaged in caregiving may be due to several factors, such as: (a) the high social acceptance of MD, resulting in the valorization of the caregiver role; (b) the awareness of poor alternatives to family care, which become increasingly necessary while treating a long-term illness [39]; (c) the availability of welfare benefits due to family assistance [29]; (d) the innate love and sense of responsibility toward a child affected by a disabling illness with an unfavorable outcome [40, 41]; and (e) the process of adapting to an illness in a loved one, which involves lowered use of emotion-focused coping strategies, such as avoidance, which proves ineffective for managing long-term stress [35, 42, 43]. Among the 26 (5 %) caregivers stating that they had considered parting from their patients, 23 (92 %) were mothers and 16 (61 %) were employed, and they were relatives of patients with average duration of illness of 10.1 (6.9 SD) years. Furthermore, they were relatives of patients with higher levels of dependency (69.1 ± 31.0 versus 54.4 ± 34.1, *F* = 5.4, df 1,500; *p* < 0.02), and they received lower psychological support from their friends (2.7 ± 0.6 versus 2.3 ± 0.3, *F* = 6.5, df 1,500; *p* < 0.01) and/or partners (3.1 ± 0.7 versus 2.7 ± 0.7, *F* = 5.5, df 1,454; *p* < 0.02), compared with relatives who did not report these feelings.

Key relatives who reported a higher objective burden were more convinced that the situation had positive consequences for their lives. This finding shows that, when relatives feel they can manage the practical difficulties of caring for patients with MD, their tolerance threshold for stress is not exceeded, thus they can consider positive aspects of caregiving despite the burden [16, 19]. Key relatives’ long-term adaptation to caring for patients with MD was also confirmed by statistics showing that, the longer key relatives have been involved in caregiving, the higher the positive evaluation they give to the experience.

Though this study does not examine caregiving for later, more severe stages of MD, it is likely that in such cases, when tasks become too demanding for key relatives, their perceptions of positive aspects may decline, while the psychological consequences of caregiving may increase [9, 39].

Our study further finds that key relatives who had more positive attitudes toward their patients were more likely to identify the positive aspects of the situation. This finding suggests the importance of helping key relatives to identify “the person beyond the illness” as a strategy to valorize their lived experience and to support their patient’s adaptation to the illness [31].

In this study, intrafamily factors were shown to be very significant and related to key relatives’ acknowledgement of positive aspects of caregiving. In particular, key relatives who felt supported by their spouses or partners were more likely to identify positive aspects of caregiving. This finding is in line with data from previous studies [7, 26, 41] in which the occurrence of a severe disease was found to strengthen the bond between the parents of child patients. Strengthened partnership bonds generally appear in our study, in which more than 70 % of key relatives considered themselves totally understood and helped by their partner.

Psychological support from social networks and professionals were both related to key relatives’ perceptions of caregiving as a positive experience. These results support research evidence that suggest that social networks are critical factors in reducing the detrimental effects of stress and serve as a buffer between coping with an event and stress [26, 34, 44]. This finding was also supported by the inverse correlation found between key relatives’ sense of family burden and sense of support from the social network [32]. Furthermore, though investigation of the impact of social resources on caregiver’s negative feelings was not among the aims of this study, it is likely that relatives with low social support and those unemployed are potentially at risk to perceive anger and injustice.

### Methodological considerations

To our knowledge, this is the first study of the condition of families of young patients with MD (1) to be performed on a large, national sample of key relatives of patients with different types of severe MD; (2) to use well-validated assessment tools already available in several languages; (3) to examine the positive aspects of caregiving in relation to burden of care, as well as key relatives’ personal factors, or attitudes toward the patient, and social and professional resources; and (4) to examine the positive experience of caregiving by means of quantitative measures and key relatives’ subjective descriptions.

Due to its cross-sectional design, this study does not allow inferences regarding the evolution of positive aspects over time or whether external resources and attitudes toward the patient influence the key relative’s perception of positive aspects or vice versa.

The survey focused on the experience lived by relatives of patients with DMD, BMD or LGMD aged 4–25 years. Therefore, its findings cannot be generalized to families of older patients or patients suffering from other types of MD. These aspects will be specifically addressed in further studies, which are now in the planning stage.

### Practical implications

The results of this study may be useful for clinicians engaged with families of persons with MD to better understand the complexity of the caregiving process and to learn how to support caregiving families. Clinicians and other professionals should help caregivers to develop not just practical but also cognitive competencies to deal with providing care for MD patients and to identify the benefits of the caregiving situation without denying its difficulties. In particular, professionals should: (a) provide families with information on the patient’s disease, according to a step-by-step approach; (b) teach relatives to reinforce their problem-oriented coping strategies; and (c) invite the relatives to share their experiences with other families through involvement with family associations and self-help groups [45].

Furthermore, clinicians themselves should develop positive attitudes towards family caregiving. This is not always the case, as reported by Green [16], a mother of a patient with cerebral palsy and a researcher, who stated that “...parents who hold positive attitudes toward raising a child with disability are often pathologized as being unrealistic, failing to accept their “tragic” circumstances, or being “in denial” of their children problems. Pathologizing, and thus discouraging, parental ability to find benefits in having a child with disability is potentially very problematic for parents.... If parents of children with disability are repeatedly discouraged from finding and acknowledging the positive aspects of caregiving, they may be denied the potentially positive consequences of doing so.”

### Future studies

Further investigations are needed to specifically explore the following aspects of caregiving in MD: (a) key relatives’ adaptation at different stages of the illness, (b) the experience of caregiving in other relatives of the same family, (c) the effects of relatives’ attitudes on patients’ perceptions of their own experiences with illness, (d) the effects of supportive interventions to improve relatives’ coping strategies, and (e) the influence of acceptance and awareness of the disease on the well-being of relatives and patients.

Forthcoming studies by our research group on a larger sample of older patients with MD will provide a model for the negative/positive feelings in relation to progression of the illness, and a profile of the psychological perception of the disease over time.
